# Assessment of Quality of Life in Patients with Chronic Anal Fissures: A 1-Year Follow-Up Study before and after Botulinum Toxin (Botox) Injection

**DOI:** 10.3390/jcm13020316

**Published:** 2024-01-05

**Authors:** Sonia-Roxana Burtic, Luca Castiglione, Marius Murariu, Ovidiu Rosca, Catalin Dumitru, Octavian Neagoe

**Affiliations:** 1 Doctoral School, “Victor Babes” University of Medicine and Pharmacy Timisoara, Eftimie Murgu Square 2, 300041 Timisoara, Romania; dr.soniaburtic@umft.ro; 2 Department II, Discipline of Medical Communication, “Victor Babes” University of Medicine and Pharmacy Timisoara, Eftimie Murgu Square 2, 300041 Timisoara, Romania; 3 Department of General Surgery, “Victor Babes” University of Medicine and Pharmacy Timisoara, Eftimie Murgu Square 2, 300041 Timisoara, Romania; murariu.marius@umft.ro; 4 Department of Infectious Diseases, “Victor Babes” University of Medicine and Pharmacy Timisoara, Eftimie Murgu Square 2, 300041 Timisoara, Romania; rosca.ovidiu@umft.ro; 5 Department of Obstetrics and Gynecology, “Victor Babes” University of Medicine and Pharmacy Timisoara, Eftimie Murgu Square 2, 300041 Timisoara, Romania; dumitru.catalin@umft.ro; 6 Second Discipline of Surgical Semiology, First Department of Surgery, “Victor Babes” University of Medicine and Pharmacy Timisoara, Eftimie Murgu Square 2, 300041 Timisoara, Romania

**Keywords:** anal fissure, Botox, quality of life, longitudinal studies

## Abstract

This longitudinal study aimed to assess the quality of life in patients with anal fissures treated with botulinum toxin (Botox) injections over a one-year period. The study hypothesized that Botox injections would significantly improve quality of life and that these improvements would be sustained over a year. Conducted as a cross-sectional study, it assessed adults diagnosed with chronic anal fissures unresponsive to conventional treatments. Participants received 25 U of Botox in two sessions and their quality of life was assessed using the WHOQOL-BREF, COPE-60, Hospital Anxiety and Depression Scale (HADS), and SF-36 surveys. Data were collected at baseline six months and one year post-treatment. The study involved 113 patients, with a mean age of 38.1 years. Significant improvements were observed in the WHOQOL-BREF scores across all domains from baseline to 12 months (physical domain: 49.4 ± 10.5 to 70.2 ± 10.6, *p* < 0.001; mental domain: 34.8 ± 11.2 to 61.9 ± 11.5, *p* < 0.001). SF-36 scores also showed significant enhancements in physical and mental health components (physical: 44.3 ± 7.5 to 56.9 ± 5.9, *p* < 0.001; mental: 41.1 ± 7.2 to 54.4 ± 6.3, *p* < 0.001). Additionally, significant improvements were noted in patient perception on quality of life from the perspective of various aspects including physical discomfort, pain management, and mood and emotional well-being. The study demonstrated that Botox injections significantly improved the quality of life in patients with chronic anal fissures, with sustained benefits observed over a year. These findings suggest Botox as an effective treatment modality for enhancing life quality in patients with this condition, highlighting the potential for broader applications in managing chronic anal fissures.

## 1. Introduction

Anal fissures, characterized by small tears in the mucosal lining of the anus, are a prevalent yet debilitating condition causing severe pain and discomfort, particularly during bowel movements [1,2]. The etiology of anal fissures is multifactorial, often associated with increased sphincter tone and ischemia of the anoderm [3]. Despite their small size, these fissures can significantly impact patients’ quality of life, leading to chronic pain, bleeding, and, in some cases, fear of defecation due to anticipated pain [4,5]. This condition, prevalent in both sexes and across various age groups, poses a considerable challenge in proctology [6].

The conventional treatment for chronic anal fissures includes dietary fiber supplements, stool softeners, and topical analgesics [7]. However, these treatments are often only partially effective and fail to provide long-term relief. Despite being effective, surgical options, such as lateral internal sphincterotomy, carry risks of incontinence and other complications [8,9]. Consequently, there is a growing interest in minimally invasive treatments that can offer both efficacy and safety. Botulinum toxin (Botox), a neurotoxin known for its muscle-relaxing properties, has emerged as a promising alternative [10].

Botulinum toxin’s mechanism of action involves the inhibition of acetylcholine release at the neuromuscular junction, resulting in temporary muscle paralysis [11]. In the context of anal fissures, Botox injections into the anal sphincter reduce sphincter spasms, increase blood flow to the fissure site, and facilitate healing [12]. Several studies have demonstrated the efficacy of Botox in healing fissures and alleviating symptoms, with a lower risk of incontinence compared with surgical interventions [13,14].

Despite the growing body of literature on the effectiveness of Botox in treating anal fissures, there remains a gap in understanding its long-term impact on patients’ quality of life [15]. Quality of life, a multidimensional construct, encompasses not only physical well-being but also psychological, social, and functional aspects [16]. In the context of anal fissures, the recurrent pain and discomfort can lead to anxiety, social isolation, and diminished overall life satisfaction [17]. Therefore, evaluating quality of life is crucial for comprehensively understanding the benefits and limitations of Botox as a treatment modality.

The existing literature predominantly focuses on the clinical efficacy of Botox in terms of healing rates and symptom relief. However, there is a scarcity of longitudinal studies that examine the sustained impact of this treatment on quality of life over an extended period. Most studies provide a snapshot of patient outcomes in the immediate or short-term post-treatment period, leaving the long-term effects relatively unexplored [18,19].

This study aims to conduct a 1-year longitudinal analysis of quality of life in patients with anal fissures treated with botulinum toxin injections. The hypotheses driving this research are twofold: firstly, that Botox injections significantly improve quality of life in patients with anal fissures and, secondly, that these improvements are sustained over a one-year period post-treatment. The study’s objectives include evaluating the changes in physical symptoms, psychological well-being, social functioning, and overall life satisfaction pre- and post-Botox treatment, thereby providing a comprehensive understanding of its long-term efficacy in managing anal fissures.

## 2. Materials and Methods

### 2.1. Research Design and Ethical Considerations

This longitudinal, observational study was conducted to assess the impact of botulinum toxin (Botox, ALLERGAN^®^, Irvine, CA, USA) injections on the quality of life in patients with anal fissures over a one-year period. Patients were recruited after admission to the general surgery clinic at the Pius Brinzeu Clinical Emergency Hospital in Timisoara, Romania, affiliated with the Victor Babes University of Medicine and Pharmacy, Timisoara. Adhering to the strictest ethical standards, the research was approved by the Local Commission of Ethics for Scientific Research, which is in alignment with the EU GCP Directives 2005/28/EC, ICH guidelines, and the principles specified in the Declaration of Helsinki. All participants provided informed consent, with confidentiality and privacy strictly maintained.

### 2.2. Inclusion and Exclusion Criteria

**Figure 1:**
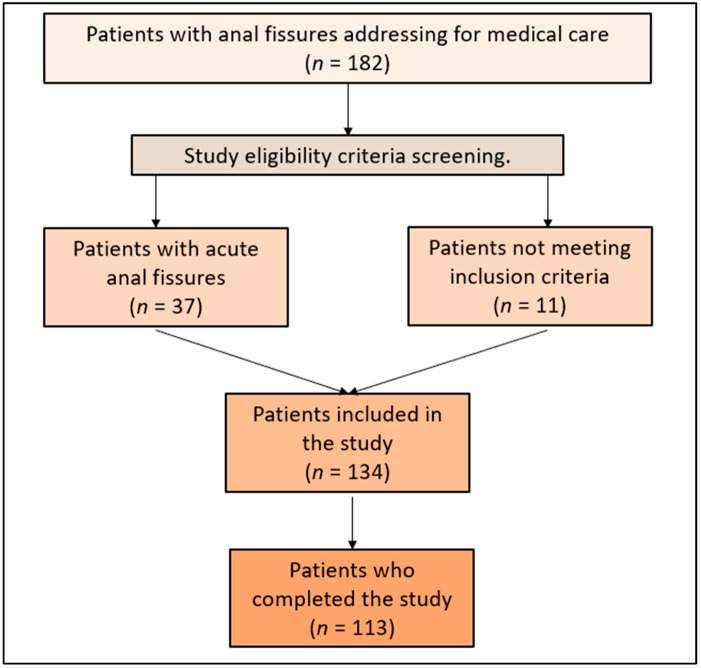
Study flowchart.

Participants were specifically recruited for the current study. The study included adult patients (aged 18 years and older) diagnosed with chronic anal fissures who had not responded to conventional treatments such as topical nitroglycerin, calcium channel blockers, or dietary fiber supplementation. Patients were required to have a documented history of the condition for at least six months prior to enrollment. Exclusion criteria were comprehensive, with individuals with previous anal surgery, acute anal fissures, co-existing colorectal conditions (e.g., Crohn’s disease and ulcerative colitis), immunocompromised states, pregnancy, lactation, and known hypersensitivity to botulinum toxin all being excluded. Patients receiving other treatments for anal fissures within six months before the study’s commencement were also ineligible, as presented in the study flowchart (Figure 1).

### 2.3. Variables and Procedures

The longitudinal assessment spanned a 12-month period post-fissure treatment. The variables assessed included the following points: age, age category, sex, area of residence, marital status, comorbidities, disease duration, anal fissure position, previous treatment, survey domain scores, and disease outcomes. The surveys were distributed online, with a response rate of 86%, and all data collected were anonymized in accordance with the EU GDPR requirements.

Eligible patients received Botox injections administered under controlled conditions by experienced proctologists. Dosages and techniques were employed based on the best current practices [20], with a 25 U dose administered to all patients as well as two injections per session for two sessions that were six months apart. Post-injection, patients were monitored for immediate side effects and given instructions for post-treatment care and symptom reporting.

The injections were carefully administered into the internal sphincter muscle at the 3 and 9 o’clock positions (lateral sites) for one group and at the 6 and 12 o’clock positions (anterior and posterior regions) for the other group in the lithotomy position. This method was based on findings suggesting that injections at the 6 and 12 o’clock positions may offer advantages in terms of post-operative pain and early complication rates. The depth of the injections was adjusted according to standard proctological practices to ensure the optimal delivery of the toxin while minimizing potential side effects [21].

### 2.4. Surveys Employed

To capture a comprehensive understanding of the participants’ experiences, multiple established tools were utilized. The WHOQOL-BREF [22], a 26-item questionnaire, was employed to evaluate the overall quality of life. The WHOQOL-BREF has been found to have good to excellent psychometric properties, performing well in preliminary tests of validity and reliability. Its robustness and cross-cultural applicability has been shown through analysis using data from 23 countries with over 11,830 participants [23]. The COPE-60 inventory [24] was also introduced in the current study to assess the diverse coping strategies adopted by the patients before and after anal fissure treatment. Regarding mental health concerns, the Hospital Anxiety and Depression Scale (HADS) [25] combining a total of 14 questions was employed to assess the presence and severity of anxiety and depressive symptoms among the participants and had composite reliability values between 0.65 and 0.78, indicating good reliability [26].

The COPE-60 has strong internal consistency, with Cronbach’s alpha scores ranging from 0.80 to 0.90 and with good validity and reliability across various scenarios [27]. The inventory is broken down into different subscales, each representing different coping strategies (disengagement, engagement, emotion-focused, and problem-focused)

(a) Disengagement. This is a form of avoidance coping where individuals detach themselves from the stressor or the associated emotions. A higher score in this subscale might indicate that a person tends to avoid dealing with the stressor.
(b) Engagement. This is a coping strategy approach where individuals actively confront and engage with the stressor. A higher score here might mean that the individual tends to address stressors head-on.
(c) Emotion-Focused. This type of coping concerns managing emotional distress rather than the actual problem or situation causing the distress. Higher scores indicate that the individual frequently uses emotion-focused strategies like seeking emotional support or expressing feelings.
(d) Problem-Focused. This strategy is about directly addressing the problem. Higher scores on this subscale mean that the individual prefers to take direct actions to resolve the stressor.

The unstandardized survey was conceived to allow for a more detailed understanding of the patients’ day to day lives with chronic anal fissures using the following questions answered on a scale from 1 to 10:

1. Physical discomfort: on a scale of 1 to 10, how would you rate your level of physical discomfort due to anal fissures? (1 being no discomfort, 10 being severe discomfort).
2. Pain relief: how effectively are you able to manage the pain associated with your condition? (1 being not effectively at all, 10 being extremely effectively).
3. Impact on daily activities: to what extent do anal fissures impact your daily activities? (1 being no impact, 10 being a significant impact).
4. Mood and emotional well-being: how would you rate your overall mood and emotional well-being? (1 being very poor, 10 being excellent).
5. Social engagement: how comfortable are you engaging in social activities? (1 being very uncomfortable, 10 being very comfortable).
6. Personal relationships: how have your personal relationships (with family, friends, etc.) been affected by your condition? (1 being not affected at all, 10 being significantly affected).
7. Work or educational activities: how has your condition affected your ability to work or engage in educational activities? (1 being no impact, 10 being a significant impact).
8. Bowel movement comfort: how comfortable are bowel movements since receiving treatment? (1 being very uncomfortable, 10 being very comfortable).
9. Sleep quality: how would you rate the quality of your sleep since being diagnosed with anal fissures? (1 being very poor, 10 being excellent).
10. Overall quality of life: how would you rate your overall quality of life since receiving the Botox treatment? (1 being very poor, 10 being excellent).

Participants were given the aforementioned surveys upon their admission (baseline) and at pre-defined intervals of 1-month post-intervention, 6 months post-discharge and at 12 months after the first Botox injection. This structured approach ensured consistent tracking and assessment.

### 2.5. Statistical Analysis

Data management and analysis were conducted utilizing the statistical software of SPSS version 26.0 (SPSS Inc., Chicago, IL, USA). The sample size was calculated based on a convenience sampling method, with a minimum of 88 respondents at a 95% confidence level and 10% margin of error. Continuous variables were represented through a mean ± standard deviation (SD), while categorical variables were expressed in terms of frequencies and percentages. To analyze the changes between more than two means of continuous variables, the ANOVA test was utilized. The Chi-square test was utilized for the categorical variables. A *p*-value threshold of less than 0.05 was set for statistical significance. All results were double-checked to ensure accuracy and reliability.

## 3. Results

Table 1 outlines the background characteristics of the 113 patients involved in the study. The mean age of the participants was 38.1 years, while the age distribution showed a predominance of middle-aged participants (40–65 years) who constituted 77.9% (88 patients) of the study population, while young adults (18–39 years) made up 22.1% (25 patients). In terms of sex distribution, there were 54.0% male patients and 46.0% female patients. Most of the participants resided in urban areas (69.9%). Regarding marital status, a majority of the participants were in a relationship or married, accounting for 70.8% (80 patients).

A Charlson Comorbidity Index (CCI) greater than two was observed in 18.6% of patients. The average duration of the disease among the participants was 10.3 months. Regarding the location of the anal fissure, 79.6% (90 patients) had posterior fissures and 20.4% (23 patients) had anterior fissures. Prior to the study, 66.4% (75 patients) of the participants had received medical treatment and 33.6% (38 patients) had undergone surgical interventions.

The outcomes post-Botox injection showed that 78.8% (89 patients) experienced the healing of the fissure, while there was a recurrence in 14.2% (16 patients) and incontinence reported in 7.1% (8 patients) of the cases during the study period.

In the physical domain, the mean score improved significantly from 49.4 before treatment to 68.7 one month after treatment. This improvement was sustained at 6 months (75.3 ± 8.4) but slightly decreased at 12 months (70.2 ± 10.6), indicating the long-term positive impact of Botox injections on physical well-being (*p*-value < 0.001). The mental domain showed a similar trend, with an initial score of 34.8 that increased to 60.5 after treatment. This improvement was even more pronounced at 6 months (68.4 ± 9.1) and remained elevated at 12 months (61.9 ± 11.5), though with a slight decrease from the 6-month mark.

**Figure 2:**
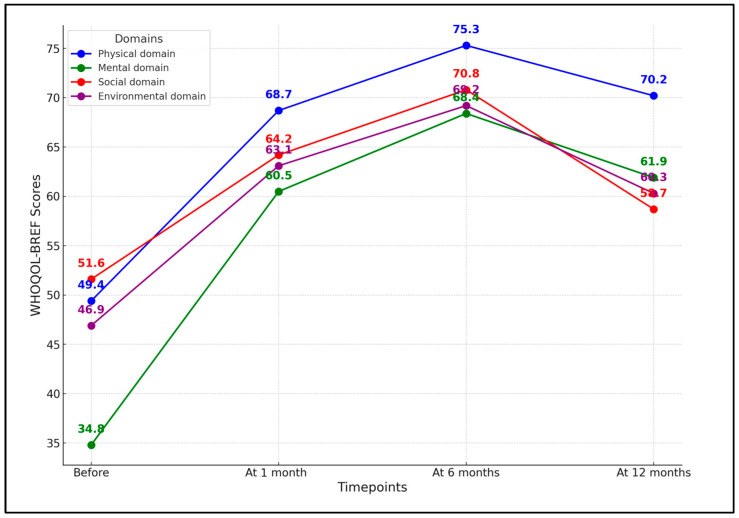
Trends in WHOQOL-BREF domains over 1 year following initial Botox treatment.

In the social domain, the initial score of 51.6 ± 12.6 increased to 64.2 ± 11.3 after treatment, peaked at 70.8 ± 10.5 at 6 months, and then decreased to 58.7 ± 9.4 at 12 months. Despite the decrease, the 12-month score was still higher than the pre-treatment score and the changes across the time points were statistically significant (*p* < 0.001). Finally, the environmental domain showed a rise from 46.9 ± 13.1 before treatment to 63.1 ± 12.7 after treatment. This score further increased to 69.2 ± 11.6 at 6 months and then dropped to 60.3 ± 13.3 at 12 months. Like the other domains, the *p*-value was <0.001, indicating significant improvements, as presented in Table 2 and Figure 2.

Initially, a high percentage of patients (72.1%) were identified with using disengagement as a coping strategy before treatment. Post-Botox injection, there was a significant reduction in the use of disengagement strategies, dropping to 30.2% one month after treatment, further decreasing slightly to 25.4% at 6 months, and then slightly increasing to 33.7% at 12 months.

Conversely, engagement, as a coping strategy, was initially lower (41.8%) before treatment. This percentage increased to 56.2% after treatment, peaked at 60.3% at 6 months, and then slightly decreased to 51.4% at 12 months (*p*-value = 0.034). The use of emotion-focused coping strategies showed a pattern similar to disengagement. Though initially high at 79.1%, it significantly decreased to 37.5% after treatment, slightly dropped to 35.6% at 6 months, and then increased to 40.8% at 12 months (*p*-value < 0.001).

**Figure 3:**
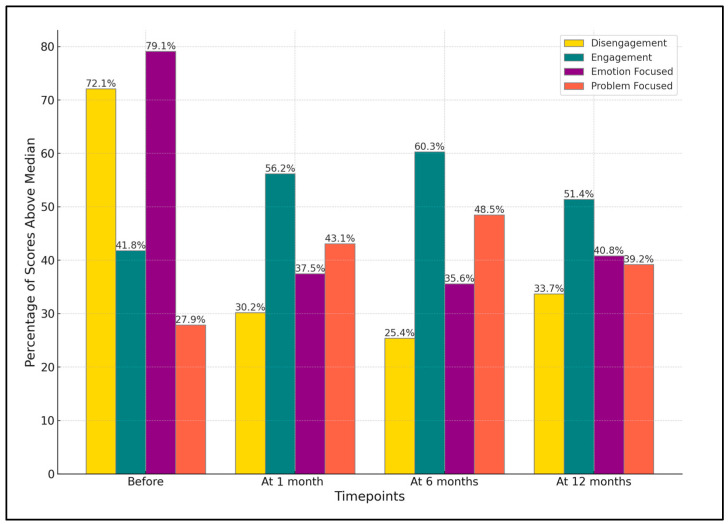
Evolution of coping strategies post-Botox treatment over one year.

Problem-focused coping showed an inverse pattern. Initially, only 27.9% of patients used problem-focused strategies, but this increased to 43.1% after treatment, peaked at 48.5% at 6 months, and then slightly decreased to 39.2% at 12 months (*p*-value = 0.013), as described in Table 3 and Figure 3.

**Figure 4:**
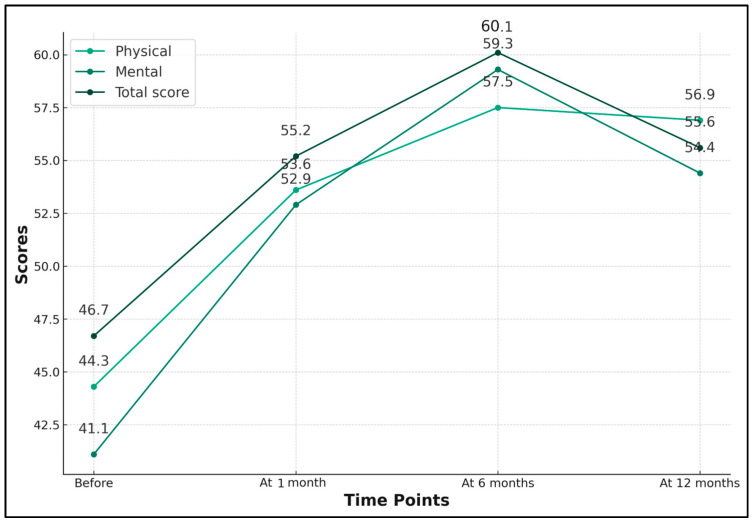
Longitudinal SF-36 health status scores after Botox treatment for anal fissures.

The mean score for physical health was 44.3 (±7.5), which showed a notable improvement to 53.6 (±6.8) 1 month after treatment and to 56.9 (±5.9) after 12 months (*p*-value < 0.001). This indicates that the improvements in physical health status post-Botox treatment were statistically significant and were sustained over the one-year follow-up period. For mental health, the initial mean score was 41.1 (±7.2). Post-treatment, this score improved to 52.9 (±6.6). A further increase was observed at 6 months, with the score reaching 59.3 (±6.9), before slightly reducing to 54.4 (±6.3) at 12 months. The total score, representing the overall health status and quality of life, also showed a similar trend. It improved from an initial mean of 46.7 (±7.8) to 55.2 (±7.0) post-treatment, peaked at 60.1 (±5.3) at 6 months, and then slightly decreased to 55.6 (±6.5) at 12 months (*p*-value < 0.001), as seen in Table 4 and Figure 4.

For physical discomfort, the mean score decreased significantly from 8.2 before treatment to 3.5 after treatment, further improving at 6 months and slightly increasing again at 12 months to 4.1. In terms of pain relief, scores increased significantly from 4.6 (±1.2) before treatment to 7.4 (±1.3) post-treatment, suggesting improved pain management. This score peaked at 8.1 (±1.2) at 6 months and then slightly decreased to 6.7 (±2.4) at 12 months (*p*-value < 0.001).

The impact on daily activities also showed a notable decrease in scores from 7.5 (±2.3) to 2.9 (±2.5) post-treatment, which further decreased to 2.4 (±2.6) at 6 months and slightly increased to 3.6 (±0.8) at 12 months. Mood and emotional well-being scores increased from 3.2 (±1.6) to 6.8 (±1.4) post-treatment, continued to increase to 7.5 (±1.3) at 6 months, and then decreased to 6.0 (±1.5) at 12 months (*p*-value < 0.001). Social engagement, personal relationships, work or educational activities, bowel movement comfort, sleep quality, and overall quality of life all showed similar patterns of significant improvements post-treatment, with the highest scores generally observed at 6 months, as shown in Table 5.

## 4. Discussion

One of the most significant findings of this study is the sustained improvement in quality of life across various domains, as evidenced by the longitudinal assessments using the WHOQOL-BREF, SF-36, and COPE-60 surveys. These improvements were not only statistically significant but also clinically relevant, highlighting the positive impact of Botox injections on both the physical and mental health aspects of patients suffering from chronic anal fissures. The fact that these improvements were maintained over a 12-month period post-treatment is particularly noteworthy, suggesting that Botox offers not just immediate relief but also long-term benefits. Therefore, in adherence to other study reports, Botox injections are a viable alternative to more invasive surgical methods like lateral internal sphincterotomy (LIS) that, while effective, often carry a risk of complications such as incontinence, a complication only reported by 7.1% of our patients and overall between 5% and 10% of patients in the majority of studies [27,28].

Another crucial aspect of this study is the emphasis on patient perception on quality of life, a factor often overlooked in clinical trials. The marked improvements in patient-reported outcomes such as physical discomfort, pain management, and impact on daily activities provide a more comprehensive picture of the treatment’s efficacy. This patient-centered approach is particularly valuable in conditions like anal fissures where subjective experiences play a significant role in treatment success.

The study’s patient demographic, that of predominantly middle-aged individuals, offers valuable insights into the population segment most affected by chronic anal fissures and potentially most responsive to Botox treatment. This information is vital for clinicians in tailoring treatment approaches and for future research in identifying and addressing the needs of specific patient groups.

In addition to Botox, reports suggest that the application of topical 2% diltiazem gel, a calcium channel blocker, was reported to lead to significant pain reduction within the first week preceding notable healing at six weeks, which correlate to the quality-of-life improvement in our cohort of patients [29,30]. Compared with Botox, diltiazem is another non-surgical approach for anal fissure management that demonstrates few side effects, such as headaches and pruritus ani, and minimal systemic effects, such as blood pressure changes [31].

In the context of quality of life, chronic anal fissures typically resulted in post-defecatory pain, bleeding, and irritation, significantly impacting patients’ daily lives [18]. Other studies [5,8] showed that pain adversely affected all SF-36 subscales, which was demonstrated by the significantly lower SF-36 scores in our study. Tsunoda et al. [18] suggest that that pre-treatment pain scores were closely linked to bodily pain and social functioning, whereas irritation scores correlated with vitality and mental health on the SF-36 scale. Similarly, older patients reported lower scores in physical functioning but unexpectedly higher scores in vitality. Contrary to previous studies [32], the duration of fissures was inversely associated with emotional role scores on the SF-36 scale, necessitating cautious interpretation of male/female differences in mental health outcomes. Similarly, other studies attributed a more important role to personality types regarding quality of life in patients with anal fissures, more specifically type D personality [33,34].

Our study also focused on the broader impact of anal fissures on patients’ physical, mental and emotional health before and after medical intervention. Post-treatment improvements were observed across various domains, including bodily pain, mental health, vitality, and general health. This finding contrasted with the study by Griffin et al. [8], where no significant improvement in mental health was reported, possibly due to the heterogeneity of treatments and the lack of specific quality of life assessments for each treatment group. Nevertheless, our study utilized a non-specific questionnaire to assess the quality of life in patients with anal fissures, which was also performed by Ortiz et al. [35] in their study, even though they compared the quality-of-life improvement after LIS procedure.

In our study, we observed significant improvements in the quality of life in patients with anal fissures following treatment using non-specific questionnaires like the SF-36. However, the emergence of disease-specific questionnaires, as highlighted by other authors, brings a new perspective to this field. The development and psychometric validation of the first questionnaire specifically designed to assess the impact of hemorrhoidal disease and anal fissures on quality of life, known as the HF-QoL questionnaire, represent a significant advancement [19]. This specialized tool demonstrated increased scores correlating with symptom severity, such as pain and bleeding, and the impact on daily activities, including days off work and increased personal spending due to these disorders. This specificity in measurement underscores the need for the adoption of such targeted instruments in future research to obtain a more nuanced understanding of the impact of these conditions on patients’ lives.

Critically analyzing the findings, the HF-QoL questionnaire’s ability to correlate strongly and consistently with the physical and psychological dimensions of established quality-of-life scales (SF-12 and PGWBI) validates its effectiveness in capturing the multifaceted impact of these conditions. Interestingly, the questionnaire showed weaker correlations with the disease-specific dimensions of defecation and sexuality, suggesting its unique capacity to quantify the general and specific impacts of these disorders on the affected body region [19]. Notably, patients with anal fissures reported higher sub-scores in the defecation dimension of the HF-QoL, indicating a more significant impact on quality of life compared with those with hemorrhoids. This aligns with the higher pain scores observed in patients with fissures and validates the greater pain intensity and symptom burden associated with anal fissures that proctologists often witness in clinical practice. Our study, while pivotal in highlighting the improvements post-treatment, could benefit from integrating such disease-specific tools in order to provide a more comprehensive and precise assessment of patient outcomes.

Nevertheless, considering the recurrence of anal fissures, further research is needed to evaluate the efficacy of additional Botox injections. Studies indicated that repeat multiple injections were used without the need for sphincterotomy, suggesting a potential role in managing recurrent cases [21,35]. This suggests that for patients with recurrent anal fissures, additional Botox injections might be a beneficial avenue to explore.

This study marks a significant advancement in the field by conducting a comprehensive one-year longitudinal analysis on a unique Romanian cohort, a demographic previously unexplored in this context. By employing a diverse array of instruments to meticulously assess both physical and mental quality-of-life facets, our approach offers a unique in-depth exploration of the sustained impact of botulinum toxin injections on patients with anal fissures. The extended follow-up period provides a better perspective on the long-term efficacy and psychological effects of the treatment, setting a new standard for future research in this area.

The current study, while offering valuable insights into the efficacy of botulinum toxin injections for chronic anal fissures, has several limitations. Its observational design without a control group limits the ability to definitively attribute improvements solely to the treatment. The convenience sampling method and the specific patient population from a single clinical setting in Romania may affect the generalizability of the findings. Additionally, the reliance on self-reported measures introduces subjectivity and potential biases into the assessment of quality of life. Moreover, the exclusion of patients with previous anal surgeries or co-existing conditions may limit the applicability of the results to the broader patient population.

Our study did not assess patients’ bowel habits and stool forms, which are critical factors in anal fissure treatment and can significantly impact outcomes and quality of life. The absence of these data is a limitation of our research as it prevents a comprehensive understanding of how these factors might have influenced the treatment efficacy and patient-reported improvements. Moreover, constipation is a recognized risk factor for the development of anal fissures; however, our study did not address whether Botox injections provide any relief for constipation symptoms. This constitutes a limitation of our research as understanding the relationship between Botox treatment and constipation symptoms could further clarify the comprehensive benefits and limitations of this therapy for anal fissures. A notable limitation of our study is the lack of data collection regarding anal sex, which may serve as a potential confounder in understanding the comprehensive impact of anal fissures. Lastly, the study’s geographic specificity might introduce regional biases, affecting the universality of its conclusions. These factors should be carefully considered when interpreting the study’s outcomes and in the design of future research.

## 5. Conclusions

In conclusion, the study conclusively demonstrates that botulinum toxin (Botox) injections significantly improve the quality of life in patients with chronic anal fissures, with these benefits persisting over a one-year period. The enhancements were particularly notable from the perspective of both physical and mental health aspects as evidenced by marked improvements across various domains of WHOQOL-BREF and SF-36 surveys. These findings highlight the effectiveness of Botox not only in alleviating the physical symptoms of anal fissures but also in substantially enhancing patients’ mental and social well-being, thereby supporting its use as a long-term treatment strategy for this condition.

## Figures and Tables

**Table 1:** Baseline characteristics of patients with chronic anal fissures.

Variables	*n* = 113	%
Age (mean ± SD)	38.1 ± 12.6	-
Age category		
Young adults (18–39 years)	25	22.1%
Middle age (40–65 years)	88	77.9%
Sex		
Male	61	54.0%
Female	52	46.0%
Area of residence		
Urban	79	69.9%
Rural	34	30.1%
Marital Status		
Single/Divorced	33	29.2%
In a relationship/Married	80	70.8%
CCI > 2	21	18.6%
Disease duration, months (mean ± SD)	10.3 ± 15.5	-
Location		
Anterior	23	20.4%
Posterior	90	79.6%
Previous treatment		
Medical	75	66.4%
Surgical	38	33.6%
Outcomes		
Healing	89	78.8%
Recurrence	16	14.2%
Incontinence	8	7.1%

SD—standard deviation; CCI—Charlson Comorbidity Index.

**Table 2:** Changes in WHOQOL-BREF scores before, 1 month, 6 months, and 12 months after the first Botox session.

WHOQOL-BREF (Mean ± SD)	Before	After	At 6 Months	At 12 Months	*p*-Value *
Physical domain	49.4 ± 10.5	68.7 ± 9.8	75.3 ± 8.4	70.2 ± 10.6	<0.001
Mental domain	34.8 ± 11.2	60.5 ± 10.2	68.4 ± 9.1	61.9 ± 11.5	<0.001
Social domain	51.6 ± 12.6	64.2 ± 11.3	70.8 ± 10.5	58.7 ± 9.4	<0.001
Environmental domain	46.9 ± 13.1	63.1 ± 12.7	69.2 ± 11.6	60.3 ± 13.3	<0.001

*—ANOVA test; SD—standard deviation; WHOQOL-BREF—brief version of the World Health Organization’s quality-of-life survey (higher scores indicate better quality of life).

**Table 3:** Coping strategies as measured using the COPE-60 before, 1 month, 6 months, and 12 months after the first Botox session.

Variables (% of Scores above Median)	Before	After	At 6 Months	At 12 Months	*p*-Value *
Disengagement	72.1%	30.2%	25.4%	33.7%	<0.001
Engagement	41.8%	56.2%	60.3%	51.4%	0.034
Emotion-Focused	79.1%	37.5%	35.6%	40.8%	<0.001
Problem-Focused	27.9%	43.1%	48.5%	39.2%	0.013

*—ANOVA test; COPE—coping orientation to problems experienced inventory (higher scores indicate that patients are more likely to use a certain domain of coping strategies).

**Table 4:** SF-36 health survey results at baseline, 1 Month, 6 Months, and 12 Months after the first Botox session.

SF-36 (Mean ± SD)	Before	After	At 6 Months	At 12 Months	*p*-Value *
Physical	44.3 ± 7.5	53.6 ± 6.8	57.5 ± 5.7	56.9 ± 5.9	<0.001
Mental	41.1 ± 7.2	52.9 ± 6.6	59.3 ± 6.9	54.4 ± 6.3	<0.001
Total score	46.7 ± 7.8	55.2 ± 7.0	60.1 ± 5.3	55.6 ± 6.5	<0.001

*—ANOVA test; SD—standard deviation; SF-36—short-form survey (higher scores indicate better health status and quality of life).

**Table 5:** Patient-reported quality of life measures pre-treatment and at 1, 6, and 12 months after the first Botox session.

Questions (1–10)	Before	After	At 6 Months	At 12 Months	*p*-Value *
Physical discomfort	8.2 ± 3.1	3.5 ± 1.9	2.8 ± 1.3	4.1 ± 2.5	<0.001
Pain relief	4.6 ± 1.2	7.4 ± 1.3	8.1 ± 1.2	6.7 ± 2.4	<0.001
Impact on daily activities	7.5 ± 2.3	2.9 ± 2.5	2.4 ± 2.6	3.6 ± 0.8	<0.001
Mood and emotional well-being	3.2 ± 1.6	6.8 ± 1.4	7.5 ± 1.3	6.0 ± 1.5	<0.001
Social engagement	3.1 ± 1.7	6.1 ± 1.5	7.7 ± 2.2	6.3 ± 1.6	<0.001
Personal relationships	3.4 ± 1.5	6.9 ± 1.3	7.3 ± 2.6	6.1 ± 2.8	<0.001
Work or educational activities	4.0 ± 0.9	7.2 ± 2.4	7.6 ± 1.3	6.5 ± 1.4	<0.001
Bowel movement comfort	2.3 ± 1.3	6.5 ± 1.2	8.2 ± 3.1	7.8 ± 3.3	<0.001
Sleep quality	3.8 ± 1.5	7.1 ± 3.0	7.8 ± 2.2	6.4 ± 1.5	<0.001
Overall quality of life	4.0 ± 1.4	7.3 ± 1.8	7.9 ± 1.2	6.2 ± 2.6	<0.001

*—ANOVA test.

## Data Availability

Data available on request.
